# Diagnosis stability and outcome of psychotic episodes in a Romanian group of children and adolescents

**DOI:** 10.1097/MD.0000000000030288

**Published:** 2022-08-26

**Authors:** Florina Rad, Mihaela Stancu, Lucia-Emanuela Andrei, Florentina-Ionela Linca, Alexandra Mariana Buică, Maria-Madalina Leti, Iuliana Dobrescu, Ilinca Mihailescu, Magdalena Efrim-Budisteanu

**Affiliations:** a “Prof. Dr. Alexandru Obregia” Clinical Hospital of Psychiatry, Bucharest, Romania; b “Mina Minovici” National Institute of Legal Medicine, Bucharest, Romania; c “Grigore Alexandrescu” Clinical Emergency Pediatrics Hospital, Bucharest, Romania.

**Keywords:** adolescents, children, diagnosis stability, early onset schizophrenia, psychosis

## Abstract

Studies on early onset schizophrenia are limited because of their low prevalence but the reported results stated that early onset is associated with a poorer outcome. The present research analyzed the stability rate of the psychotic-related disorders from childhood to adult life. The study was based on an observational, retrospective, descriptive analysis study. The subjects were selected from patients admitted to the pediatric psychiatry ward of “Alexandru Obregia” Psychiatry Hospital between 2009 and 2018 for a psychosis-related disorder, who were 18 years or older at the moment of data collection and who also had admissions into the adult’s psychiatry wards of the hospital. Of the 115 subjects, 93, representing 80.87% of the total, maintained a diagnosis of psychotic spectrum disorder into adulthood. The diagnosis was maintained in 82.4% of cases with onset before 13 years old and 80.6% of cases with onset after the age of 13 years of age. Of the 42 subjects who presented affective symptoms during childhood, 71.43% also presented affective symptoms into adulthood. These findings indicate an important stability rate of psychosis from childhood and adulthood and come in accordance with the theory of overlap between psychotic and affective disorders. The results underline the importance of an accurate diagnosis of early and very early onset schizophrenia (VEOS), the need for early and multimodal intervention, but also the need for long-term management of these patients and continuing research regarding psychotic-related disorders in children and adolescents.

## 1. Introduction

Schizophrenia is a severe, chronic mental health disorder that profoundly affects daily functioning and is usually firstly diagnosed in adult age, but can also be identified in children and adolescents.^[1]^ Schizophrenia with onset under the age of 13 is called very early onset schizophrenia (VEOS), a rare disorder with an estimated 1 in 10,000 prevalence.^[2]^ Schizophrenia with onset under 18 years of age is called early-onset schizophrenia (EOS) and has an estimated prevalence of 1.4 out of 10,000.^[3]^

Studies on EOS and VEOS are limited due to the low prevalence of these disorders. On the other hand, the diagnosis of schizophrenia in children and adolescents was not differentiated from the diagnosis of autism until the publication of the third edition of the diagnostic and statistical manual of mental disorders (DSM) in 1980,^[4]^ children and adolescents with schizophrenia being diagnosed according to the same criteria as adults.^[5]^ The same study, that reviewed data published between 1980 and 2012, concluded that the patients’ unfavorable outcome is proportional with the age of onset.^[5]^

Currently, the diagnosis of schizophrenia in children and adolescents is established using the same “ICD-10” or “DSM-5” diagnostic criteria as in adults; however, it is specified that these children will never reach the expected level of functioning.^[6,7]^ However, the diagnosis at an early age is difficult to establish, the clinical picture is often mixed with affective or behavioral symptoms. Moreover, pervasive disorders with psychotic decompensation can mimic the clinical picture of schizophrenia thus leading to false positive diagnoses. On the other hand, adult-specific diagnostic criteria do not capture the developmental aspects of childhood and adolescence (e.g., magical thinking or developing psychic functions) and, as a result, there is a risk of omitting the diagnosis of schizophrenia.^[8,9]^ In a study published in 2006, Remschmidt et al showed that in a group of children initially diagnosed with schizophrenia, after applying the international classification of diseases-10 (ICD-10) diagnostic criteria, only in 50% of them this diagnosis was confirmed. The rest were confirmed with emotional disorders or conduct disorders.^[10]^

The treatment in schizophrenia is a complex one and includes both pharmacological and nonpharmacological interventions (patient and family counseling, individual therapy, assistance for school and social rehabilitation, family support) and the evolution is favorable only in a low percentage of cases.^[11]^ Lally et al (2017) examined remission and recovery after the first episode of psychosis, including affective psychoses. They concluded that 7 years after the initial onset, the cumulative remission rate was 58% and the recovery rate was 38%; they also noted that recovery rates have not improved in recent years.^[12]^

Fleischhaker study, published in 2005, regarding the outcome and stability of psychotic symptoms with onset in childhood and adolescence, conclude that the evolution is less favorable: 25% can recover, 50% have occasional relapses, and 25% are severely affected.^[13]^ The same study evaluated the prognosis of 101 patients with VEOS, using the Global Assessment Scale (GAS), concluding that trajectory of symptoms and functionality on different life areas was less severe when onset is in adolescence and presence of developmental disorders or introverted personality structure are risk factors for unfavorable prognosis.^[13]^

Regarding the factors that could be considered predictors for evolution, it is considered that VEOS correlates with a low expected educational level, poor social relationships, socio-economic difficulties in adulthood, and the continuing need for psychiatric medical care.^[2]^ Several EOS/VEOS-related studies indicate that approximately 20% of children and adolescents with psychosis have an IQ < 80, a lower premorbid IQ (intelligence quotient) among young people predicts later schizophrenia diagnosis and the most affected patients are those who have associated moderate or severe intellectual disability.^[14-17]^ Schizophrenia has also been correlated with neurodevelopmental disorders, multiple studies reporting the presence of premorbid disorders in social, linguistic or motor areas and learning disabilities as predictors for unfavorable prognosis.^[18,19]^

Therefore, most studies in the literature show a less favorable prognosis of schizophrenia with onset in childhood and adolescence compared to onset in adulthood. When the disorder begins in childhood, the evolution is even less favorable.^[13]^

Our study brings to attention a pathology with a highly debilitating character, evaluates the age-dependent predictors of chronicity of psychotic symptoms, and highlights new perspectives regarding the comorbidity that can precipitate a psychotic episode in small ages. There is a growing literature that investigates the premorbid characteristics of patients with psychosis and the stability of this premorbid phenotype as the patient ages, our research adding information on the impact of comorbidities at the age of the first diagnosis, the personal and family medical factors that influence the course of the disease. According to the results that will be debated below, the family history of mental illness not only influences the predisposition to develop mental health disorders but also predicts the early ages of developing severe symptoms and the number of relapses.

The objectives of the study are:

1. To follow the evolution of children and adolescents diagnosed with psychotic-related disorders.
2. To determine the stability rate of the diagnosis of psychotic-related disorders with onset in childhood and adolescence into adulthood.
3. To identify possible factors that contribute in maintaining the diagnosis of psychotic-related disorders in adulthood.

## 2. Materials and Methods

### 2.1. Participants and data collection procedures

The participants in this study were selected using the electronic database and the medical records of the “Alexandru Obregia” Clinical Psychiatry Hospital. The following criteria must have been met for the patients to be included in the study: (a) age at first admission in the hospital <18 years (pediatric ward); (b) diagnosis of psychotic-related disorder at the time of first admission in the hospital (<18 years); (c) age >18 years at the time of data collection (2021); (d) at least one admission in the adult psychiatry ward of the same hospital (>18 years old). One hundred fifteen participants (67 males and 48 females) met the criteria and were therefore included in the study.

All the participants included in the study were diagnosed by a child and adolescent psychiatrist using the DSM-5 diagnostic criteria, with one of the following psychotic-related disorders: schizophrenia, brief psychotic disorder, severe depressive episode with psychotic symptoms or psychotic mania. Other clinical data obtained from the medical records form the pediatric psychiatric ward were the number of admissions, age at first admission, secondary diagnosis and family history of psychosis.

Following the data collection from the pediatric psychiatric ward, the medical records from the adult psychiatric ward of the 115 participants enrolled in the study were analyzed. The data used from the medical record of the participants from the adult psychiatric clinic were the number of admissions in the adult psychiatric ward and the main and secondary diagnoses.

### 2.2. Data analysis

Descriptive statistics was used to describe the number of admissions in both pediatric and adult psychiatric wards and Spearman rank correlation was used to assess the relationship between them. To have a better estimate of the number of admissions, this was divided by the number of years with the disease (the number of years that have passed between the first diagnosis and the moment of data collection). The number obtained was defined as the *Admissions/Year of disease Coefficient and* descriptive statistics was used to describe it. The total number of admissions was also divided into 2 groups (≤5 admissions, >5 admission) and *χ*² tests were performed to determine if there is an association between the total number of admissions and the onset of the disease (≤13 years of age and >13 years of age). The stability rate of the diagnosis was determined using the frequency of the psychotic-related disorders in the medical records from the adult psychiatric ward. The stability of the affective symptoms was also determined. Next, *χ*² tests were performed to assess the relationship between the stability of the diagnosis into adulthood and age of onset, as well as the relationship between the number of admissions in the adult psychiatric ward and the presence of ASD (Autism Spectrum Disorder)/Intellectual disability, the age of onset or the presence of a family history of psychosis.

Statistical analysis was performed using JASP software, SPSS software, and R software. In all the performed tests, the level of significance was set at 0.05.

## 3. Results

The subjects’ age at the moment of the study varied between 19 and 28 years old, with a mean age of 23.57 ± 2.38 years. The subjects’ age at their first admission into the pediatric psychiatry ward varied between 6 and 17 years old, with a mean age of 15.31 ± 2.00 years old. The age of the first admission was also considered the age of the onset of symptoms. Seventeen of the participants were <13 years old at the onset of the disorder (14.74%), and 98 were over 13 years of age. 4 subjects (3.48%) were <10 years old at the onset of psychotic symptoms.

**Figure 1.**
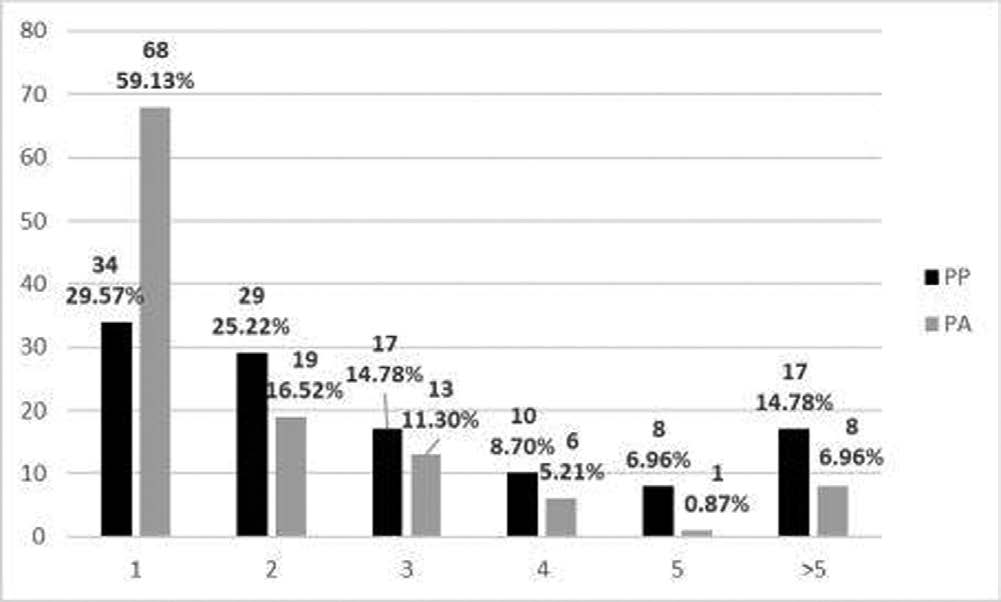
Number of admissions into the pediatric (PP) and adult’s psychiatry (PA) wards.

Thirty-four participants (29.57%) were admitted only once in the pediatric ward whereas 25 of the participants were admitted more than 5 times as children/adolescents. The maximum number of admissions per patient was 16 and the mean number of admissions was 3.17 ± 2.65 (Fig. 1).

Regarding admissions into the adult psychiatric ward, the number of admissions varied between 1 and 11, with a mean of 2.07 ± 1.94 admissions. The was no statistical correlation between the number of admissions into the pediatric and adults’ wards (*P* = .106). The Admissions/ Year of disease Coefficient varied between 0.13 and 3.66, with a mean of 0.67 ± 0.49 (mdn 0.54).

The total number of admissions (both in pediatric and adults’ wards) was 5 or less for 73 subjects (63.48%). 42 subjects had more than 5 admissions in our hospital (36.52%). There was a significant statistical correlation between the very early onset of psychotic symptoms (<13 years) and the number of admissions (*P* = .038). Of the subjects with onset after the age of 13, 67.3% had 5 or less admissions, while 58.8% with very early onset (<13 years old) had more than 5 admissions in the hospital (Table 1).

**Table 1:** Crosstab age of onset and total number of admissions.

			>5 admissions		Total
			0	1	
Debut.bf.13y	0	Count	66	32	98
		% within debut.bf.13y	67.3%	32.7%	100.0%
	1	Count	7	10	17
		% within debut.bf.13y	41.2%	58.8%	100.0%
Total		Count	73	42	115
		% within debut.bf.13y	63.5%	36.5%	100.0%

Fifty-two of the subjects (45.22%), associated comorbidity of pervasive disorder or mental retardation during childhood. Nineteen of the patients had pervasive developmental disorder and 40 had intellectual disabilities.

**Figure 2.**
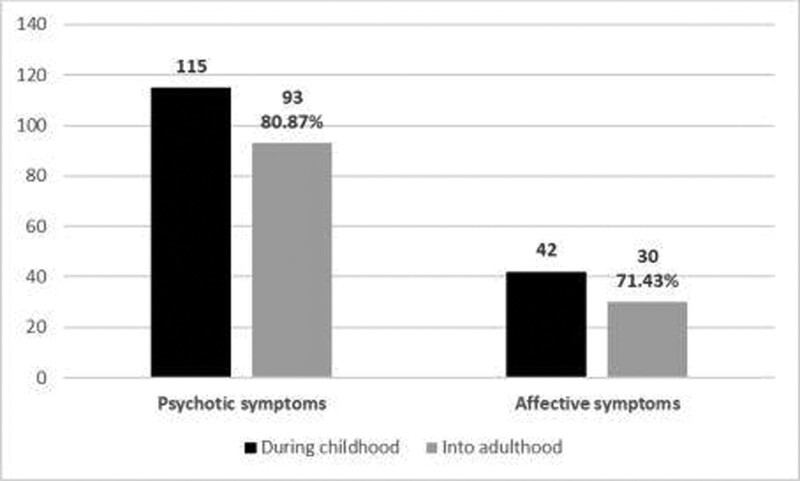
Stability of psychotic and affective symptoms into adulthood.

Regarding the diagnosis stability, we considered that all patients who, after 18 years of age, met the criteria for schizophrenia, brief psychotic disorder, severe depressive episode with psychotic symptoms, or psychotic mania maintained a psychotic-spectrum diagnosis first-expressed during childhood and adolescence. In the studied group, from the initial 115 subjects, 93 of them (80.87%), maintained one of the aforementioned diagnoses as adults. The patients who, as adults, were diagnosed with either mania or depressive disorder without psychotic elements or with a schizo-affective disorder were considered to have shifted to the affective spectrum. Of the 22 subjects who did not maintain a diagnosis of psychotic-related disorders, 8 met the diagnosis criteria for an affective disorder, while 14 had other diagnoses, including organic personality disorder, obsessive-compulsive disorder, and intellectual disability (Fig. 2).

Of the initial younger-than-18-years-old 115 psychotic patients included in the study, 42 presented affective symptoms as well. Of the 42 subjects who presented affective symptoms during childhood, 30 (representing 71.43%) also presented affective symptoms into adulthood (Fig. 2 and Table 2). At adult age, 41 patients were identified to associate affective symptomatology, with 30 of them having these elements identified since childhood (Table 2).

**Table 2:** Crosstab—presence of affective symptoms in childhood and into adulthood.

			Affective symptoms in adulthood		Total
			0	1	
Affective symptoms in childhood	0	Count	62	11	73
		% within Affective symptoms in childhood	84.9%	15.1%	100.0%
	1	Count	12	30	42
		% within Affective symptoms in childhood	28.6%	71.4%	100.0%
Total		Count	74	41	115
		% within Affective symptoms in childhood	64.3%	35.7%	100.0%

There was a significant statistical correlation between the presence of affective symptoms into childhood and the presence of the respective symptoms into adulthood (*P* < .001). 84.9% of the subject without affective symptoms in childhood did not develop the presence of affective symptoms into adulthood, while the diagnosis stability in cases of present affective symptoms was 71.4%.

Regarding the diagnosis stability in relation to the age of onset, 82.4% of cases with onset before 13 years old (VEOS) and 80.6% of cases with onset after the age of 13 maintained their diagnosis. There was no statistically significant difference between the 2 groups (*P* = .584) (Table 3).

**Table 3:** Crosstable—age of onset/stability of diagnosis into adult age.

			Adult.psychosis.diagnosis		Total
			0	1	
Debut.aft.13y	0	Count	3	14	17
		% within Debut.aft.13y	17.6%	82.4%	100.0%
	1	Count	19	79	98
		% within Debut.aft.13y	19.4%	80.6%	100.0%
Total		Count	22	93	115
		% within Debut.aft.13y	19.1%	80.9%	100.0%

**Figure 3.**
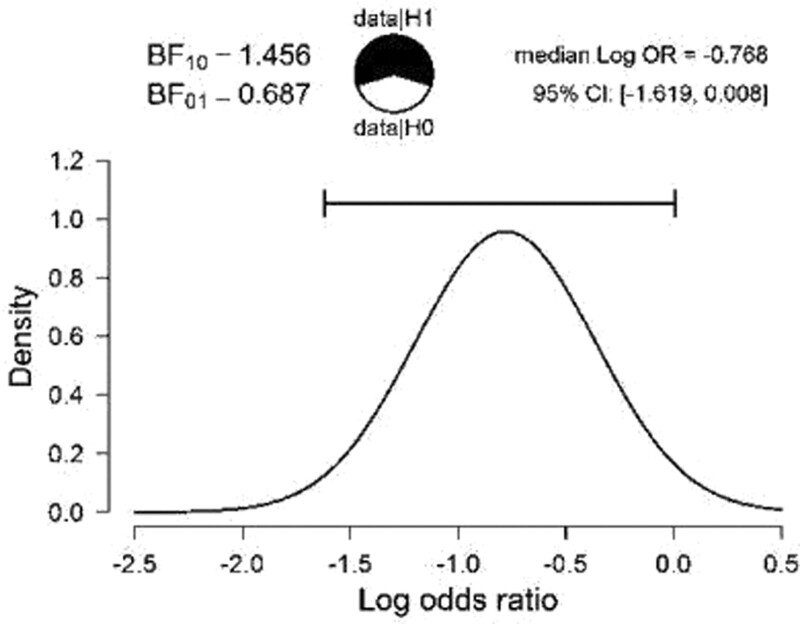
Graphical representation of the relationship between ASD, diagnosis maintained in adults and number of relapses. ASD = autism spectrum disorder.

Repeated admissions in the pediatric ward after the first psychosis episode were considered relapses and were analyzed in relationship with the diagnosis and associated factors. Ninety-eight of the participants had up to 5 relapse episodes, and 17 had over 5 relapse episodes. There are significant differences between patients who maintained their diagnosis in terms of the number of relapse episodes taking into account the existence of the diagnosis of autism (*P* < .05). Sixty-nine of the participants maintained their diagnosis, had autism, and had a large number of relapse episodes (Fig. 3 and Table 4).

**Table 4:** *χ*² tests.

	Value	df	*P*
*χ*²_ASD, diagnosis maintained in adults and number of relaps_	6.7	1	.010
*χ*² _the presence of affective elements in children, diagnosis maintained in adults_	2.69	1	.101
*χ*² _ID, diagnosis maintained in adults and number of relaps_	0.083	1	.643
*χ*² _the age of onset of the disorder and the number of relapses and the number of hospitalizations in adult psychiatry_	43.1	10	.001
*χ*² _the family psychiatric history and the number of relapses and the number of hospitalizations in adult psychiatry_	18.17	10	.040
*χ*²_ASD, diagnosis maintained in adults and age of onset_	10.1	1	.002

There were identified significant differences between patients who maintained their diagnosis in terms of the age of onset taking into account the existence of the diagnosis of autism (*P* < .05). There is a statistically significant relationship between the young age of onset of the disorder and the number of relapses and the number of hospitalizations in adult psychiatry, but also between the family psychiatric history and the number of relapses and the number of hospitalizations in adult psychiatry (*P* < .05) (Table 4).

**Figure 4.**
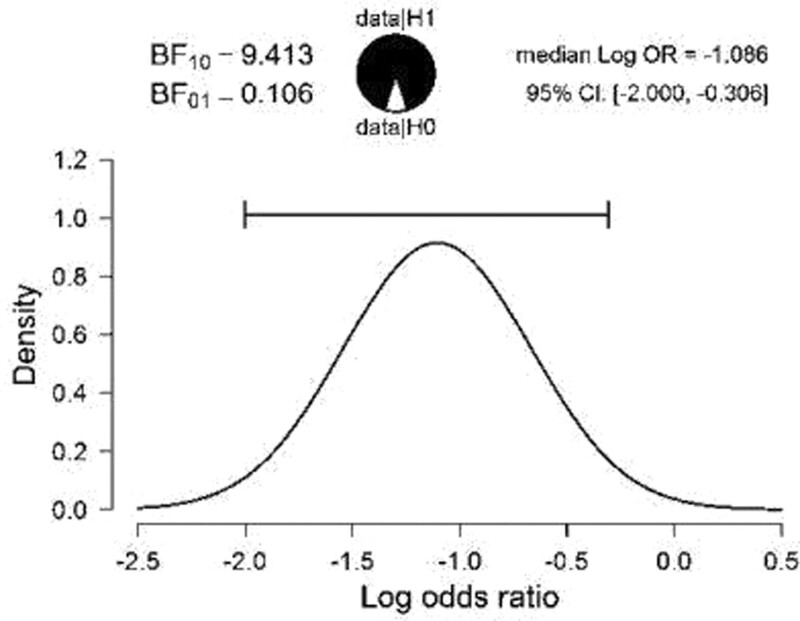
Graphical representation of the relationship between the family psychiatric history and the number of relapses and the number of hospitalizations in adult psychiatry.

Sixty-six subjects (57.40%), had a family history of psychiatric disorders. There was a significant correlation between the presence of family history and number of relapses (*P* = .040) (Table 3, Fig. 4).

## 4. Discussion

This research evaluated the taxonomic changes of psychotic-related disorder during adulthood, in a group of patients that were admitted into the pediatric psychiatry ward between 2009 and 2018 and who were subsequently admitted to the psychiatric services of the hospital after turning 18. The first result that should be discussed is that a large proportion of subjects from out-group presented onset of psychotic symptoms after the age of 13 (early onset).

Onset age is an important prognosis factor, considering that in patients with adolescent-onset psychosis, it is expected to notice a more severe clinical and functional development than in adult-onset cases.^[20,21]^ A systematic review published in 2015 demonstrated that patients with EOS that presented multiple and severe premorbid characteristics and prominent negative symptoms at initial presentation present an unfavorable long-term course.^[22]^

According to data published by Rabinowitz et al a younger age at the first hospitalization was associated with a higher probability of having more than 1 hospitalization, a longer duration of the first hospitalization, more readmissions, and more days of hospitalization per year. Among patients being admitted for the first time before the age of 17, 82.5% had more than 1 relapse with readmission. Regardless of gender, the relationship between age at first hospitalization and readmission showed a linear trend. Thus, an early onset was correlated with the severity and course of the disease as measured by relapses with readmission and appears to have a prognostic value.^[23]^

85.22% of the subjects had 5 less than admissions into the pediatric psychiatry ward after the onset, while 14.78% had more than 5 relapses. 93.04% of the subjects had 5 or less admissions into the adult psychiatry wards, with only 8 subjects being admitted more than 5 times. The total number of admissions (both in pediatric and adult wards) was 5 or less for 73 subjects, representing 63.48% of the total. Forty-two subjects, representing 36.52% had more than 5 admissions in our hospital. There was a significant statistical correlation between the very early onset of psychotic symptoms (<13 years) and the number of admissions (*P* = .038).

80.87% of our subjects maintained a diagnosis of psychotic spectrum disorder into adulthood. The stability rate of over 80% regarding psychotic symptoms is also cited in previous research papers. Several researchers have studied the stability of the diagnosis of schizophrenia with onset in childhood and adolescence. We note the research conducted by Hollis (2000) who studied the stability after 11 years of the diagnosis of schizophrenia with onset in childhood and adolescence (mean age of onset 14 years) on a group of 51 patients. The stability rate reported by Hollis was 80%.^[8]^

82.4% of cases with onset before 13 years old (VEOS) and 80.6% of cases with onset after the age of 13 maintained their diagnosis. There was no statistically significant difference between the 2 groups (*P* = .584). Remschmidt et al (2006) followed the stability of the diagnosis of EOS 42 years after the first diagnosis. According to the data obtained by them regarding the functioning of patients measured with GAS, only 16% of patients had a favorable evolution, 24% (GAS score over 70) had a moderately favorable evolution and 60% had an unfavorable evolution. Remschmidt study demonstrates a stability rate of the diagnosis of schizophrenia of 91% at 42 years after the first diagnosis. 9% of patients were classified in other diagnostic categories, namely depression or bipolar disorder.^[10]^ The stability rate of the diagnosis of schizophrenia was 100%, that of bipolar disorder was 79% and the diagnosis of major depression was 64% in a study reported by Jarbin and Knorring (2003) emphasizing the continuity of diagnoses from adolescence to adulthood.^[24]^

Chronicity of affective symptoms from childhood in a significant proportion of patients of our study is in accordance with the theory of overlap between psychotic and affective disorders, some authors suggesting a possible genetic overlap with affective disorders such as bipolar disorder or schizoaffective disorder.^[25–30]^

There are significant differences between patients who maintained their diagnosis in terms of the number of relapse episodes taking into account the existence of the diagnosis of autism (*P* < .05). Sixty-nine of the participants maintained their diagnosis, had autism, and had a large number of relapse episodes. These findings were in accordance to literature data.^[18]^ The outcome and prognosis of psychotic disorders were correlated with the presence and severity of developmental disorders, some studies suggesting that the presence and severity of these developmental deficits may actually be a premorbid phenotype for VEOS or EOS.^[19,31]^

There is also a statistically significant relationship between the young age of onset of the disorder and the number of relapses and the number of hospitalizations in adult psychiatry, but also between the family psychiatric history and the number of relapses and the number of hospitalizations in adult psychiatry (*P* < .05).

Considering aspects related to the evolution of early-onset psychoses and the stability of the diagnosis, the notion that symptoms may develop during a continuous course of the disease has gained attention in recent years, multiple genetic and environmental factors that could be incriminated in this trajectory being investigated in these studies. Among the identified factors, the family history of schizophrenia is the strongest unique indicator of the individual risk of schizophrenia.^[32]^ In addition, the risk of clinically diagnosed schizophrenia is associated with a family history of a wide range of mental disorders, with several studies finding an increased frequency of other mental health disorders in first-degree relatives of patients with schizophrenia.

Schizophrenia is a debilitating and devastating disorder, especially if it is diagnosed at an early age, during childhood or adolescence. Despite the presence of several premorbid characteristics being acknowledged, a viable premorbid phenotype is yet to be determined and thus research on the physiopathology of this disorder is ongoing, without a substantial systematically-proven target in sight. The frequency and duration of psychotic episodes take a toll on the neuropsychology, neurophysiology, and neural structure of the individual, proving early and aggressive intervention as a key element of effective care.^[33–35]^ Once a schizophrenia diagnosis is established, together with other comorbidities, clinicians should approach this condition aggressively. The therapeutic intervention should be a multi-disciplinary one, comprising of psychopharmacological elements, as well as psychotherapy and early psychosocial profiling and assistance, such as family support and psychoeducation regarding the disorder and its course, especially during the first years after diagnosis, as these elements have been proven to improve the course of the disease.^[36]^

The study has several limitations. The first one relates to the necessity to extend research on a group with a larger number of patients. The study included patients with ages 19 to 28 and we consider as future directions to investigate the dynamics of psychotic episodes further this age. Another limitation relates to the lack of data regarding presence/maintenance of the pharmacological/social intervention before and after the first diagnosis. Another limitation relates to selection of the patients exclusively from a hospital with admittance profile/acute pathologies. It was not possible to monitor the dynamics of patients who have been evaluated in outpatient services.

Future research should examine the outcome of patients older than 28 years old and replicate the results in patients that had in childhood/adolescence psychotic episodes and continued psychiatric follow-up in outpatient units.

## 5. Conclusions

This paper and its results underline the importance of an accurate diagnosis of early and VEOS, the need for early and multimodal intervention, but also the need for long-term management of these patients and continuing research regarding psychotic-related disorders in children and adolescents.

Our research brings new information regarding the factors that can influence the long-term course of EOS/VEOS, because even though is a growing interest in identifying predictors in this category of patients, there are still limited or contradictory evidence regarding these individually characteristics that can shape the maintenance/transformation of the psychotic picture.

The study results regarding the way that comorbidities, age of diagnosis, the presence of family history of mental illnesses can influence the outcome and intensity/frequency of relapses could help identify patients at risk, intensify intervention methods and monitor adherence to treatment to avoid relapses and hospitalization.

## Author contributions

**Conceptualization:** Florina Rad, Iuliana Dobrescu, Lucia-Emanuela Andrei, Magdalena Efrim-Budisteanu.

**Data curation:** Florentina-Ionela Linca, Magdalena Efrim-Budisteanu, Mihaela Stancu.

**Formal analysis:** Florentina-Ionela Linca, Ilinca Mihailescu, Mihaela Stancu.

**Investigation:** Florentina-Ionela Linca.

**Methodology:** Florentina-Ionela Linca, Maria-Madalina Leti, Mihaela Stancu.

**Project administration:** Florina Rad.

**Resources:** Florina Rad.

**Software:** Mihaela Stancu.

**Supervision:** Florina Rad.

**Validation:** Florina Rad, Magdalena Efrim-Budisteanu.

**Visualization:** Alexandra Buică, Iuliana Dobrescu, Lucia-Emanuela Andrei.

**Writing – original draft:** Alexandra Buică, Lucia-Emanuela Andrei.

**Writing – review & editing:** Alexandra Buică, Ilinca Mihailescu.

## References

[1] U.S. Department of Health and Human Services, National Institutes of Health, National Institute of Mental Health. (Updated 2021). NIMH Strategic Plan for Research (NIH Publication No. 21-MH-8082). Available at: https://www.nimh.nih.gov/sites/default/files/documents/health/publications/schizophrenia/schizophrenia.pdf.

[2] Gonthier M Lyon MA. Childhood-onset schizophrenia: an overview. [References]. Psychol Sch. 2004;41:803–11.

[3] McKenna K Gordon CT Lenane M. Looking for childhood-onset schizophrenia: the first 71 cases screened. J Am Acad Child Adolesc Psychiatry. 1994;33:636–44. 8056726 10.1097/00004583-199406000-00003

[4] American Psychiatric Association. DSM III - Diagnostic and Statistical Manual of Mental Disorders (3rd ed.). Washington, DC: The American Psychiatric Association; 1980.

[5] Clemmensen L Vernal DL Steinhausen HC. A systematic review of the long-term outcome of early onset schizophrenia. BMC Psychiatry. 2012;12:150. 22992395 10.1186/1471-244X-12-150 PMC3521197

[6] World Health Organization. ICD-10: International Statistical Classification of Diseases and Related Health Problems: Tenth Revision, 2nd ed. Geneva, Switzerland: World Health Organization, 2004.

[7] Statistical Manual of Mental Disorders (5th ed.); Washinton, DC; 2013.

[8] Hollis C. Adult outcomes of child- and adolescent-onset schizophrenia: diagnostic stability and predictive validity. Am J Psychiatry. 2000;157:1652–9. 11007720 10.1176/appi.ajp.157.10.1652

[9] Bartlett J. Childhood-onset schizophrenia: what do we really know? Health Psychol Behav Med. 2014;2:735–47. 25750815 10.1080/21642850.2014.927738 PMC4345999

[10] Remschmidt H Martin M Fleischhaker C. Forty-two-years later: the outcome of childhood-onset schizophrenia. J Neural Transm. 2007;114:505–12. 16897595 10.1007/s00702-006-0553-z

[11] Masi G Liboni F. Management of schizophrenia in children and adolescents. Drugs. 2011;71:179–208. 21275445 10.2165/11585350-000000000-00000

[12] Lally J Ajnakina O Stubbs B. Remission and recovery from first-episode psychosis in adults: systematic review and meta-analysis of long-term outcome studies. Br J Psychiatry. 2017;211:350–8. 28982659 10.1192/bjp.bp.117.201475

[13] Fleischhaker C Schulz E Tepper K. Long-term course of adolescent schizophrenia. Schizophr Bull. 2005;31:769–80. 16123530 10.1093/schbul/sbi014

[14] Bakken Trine L. Behavioural equivalents of schizophrenia in people with intellectual disability and autism spectrum disorder. A selective review. Int J Dev Disabil. 2021;67:310–7. 34567543 10.1080/20473869.2021.1925402 PMC8451634

[15] Vyas N Burke L Netherwood S. Neurocognitive profile of adolescents with early-onset schizophrenia and their unaffected siblings. World J Biol Psychiatry. 2022;10:1–12. 10.1080/15622975.2021.2023758 34989324

[16] Woodberry KA Giuliano AJ Seidman LJ. Premorbid IQ in schizophrenia: a meta-analytic review. Am J Psychiatry. 2008;165:579–87. 18413704 10.1176/appi.ajp.2008.07081242

[17] Friedlander RI Donnelly T. Early-onset psychosis in youth with intellectual disability. J Intell Disabil Res. 2004;48:540–7. 10.1111/j.1365-2788.2004.00622.x 15312054

[18] Hartopo D Kalalo R. Language disorder as a marker for schizophrenia. Asia Pac Psychiatry. 2021. 10.1111/appy.12485 34328267

[19] Driver DI Gogtay N Rapoport JL. Childhood-onset schizophrenia and early-onset schizophrenia spectrum disorders. Child Adolesc Psychiatr Clin N Am. 2020;29:71–90. 31708054 10.1016/j.chc.2019.08.017

[20] Schimmelmann BG Conus P Cotton S. Pre-treatment, baseline, and outcome differences between early-onset and adult-onset psychosis in an epidemiological cohort of 636 first-episode patients. Schizophr Res. 2007;95:1–8. 17628441 10.1016/j.schres.2007.06.004

[21] Veru F. Adolescent vs. adult onset of a first episode psychosis: impact on remission of positive and negative symptoms. Schizophr Res. 2016;174:183–8. 27102425 10.1016/j.schres.2016.03.035

[22] Díaz-Caneja CM Pina-Camacho L Rodríguez-Quiroga A. Predictors of outcome in early-onset psychosis: a systematic review. NPJ Schizophr. 2015;1:14005. 27336027 10.1038/npjschz.2014.5 PMC4849440

[23] Rabinowitz J Levine SZ Häfner H. A population based elaboration of the role of age of onset on the course of schizophrenia. Schizophr Res. 2006;88:96–101. 16962742 10.1016/j.schres.2006.07.007

[24] Jarbin H Ott Y Von Knorring AL. Adult outcome of social function in adolescent-onset schizophrenia and affective psychosis. J Am Acad Child Adolesc Psychiatry. 2003;42:176–83. 12544177 10.1097/00004583-200302000-00011

[25] Cardno AG Rijsdijk FV Sham PC. A twin study of genetic relationships between psychotic symptoms. Am J Psychiatry. 2002;159:539–45. 11925290 10.1176/appi.ajp.159.4.539

[26] Laursen TM Labouriau R Licht RW. Family history of psychiatric illness as risk factor for schizoaffective disorder. Arch Gen Psychiatry. 2005;62:841–8. 16061761 10.1001/archpsyc.62.8.841

[27] Maier W Hofgen B Zobel A. Genetic models of schizophrenia and bipolar disorder: overlapping inheritance or discrete genotypes? Eur Arch Psychiatry Clin Neurosci. 2005;255:159–66. 15995899 10.1007/s00406-005-0583-9

[28] Craddock N O’Donovan MC Owen MJ. Genes for schizophrenia and bipolar disorder? Implications for psychiatric nosology. Schizophr Bull. 2006;32:9–16. 16319375 10.1093/schbul/sbj033 PMC2632175

[29] Owen MJ Craddock N Jablensky A. The genetic deconstruction of psychosis. Schizophr Bull. 2007;33:905–11. 17551090 10.1093/schbul/sbm053 PMC2632314

[30] Lichtenstein P Yip BH Bjork C. Common genetic determinants of schizophrenia and bipolar disorder in Swedish families: a population-based study. Lancet. 2009;373:234–9. 19150704 10.1016/S0140-6736(09)60072-6 PMC3879718

[31] Budisteanu M Andrei E Linca F. Predictive factors in early onset schizophrenia. Exp Ther Med. 2020;20:210. 33149774 10.3892/etm.2020.9340 PMC7604757

[32] Mortensen PB Pedersen MG Pedersen CB. Psychiatric family history and schizophrenia risk in Denmark: which mental disorders are relevant? Psychol Med. 2010;40:201–10. 19607751 10.1017/S0033291709990419

[33] Ienciu M Romosan F Bredicean C. First episode psychosis and treatment delay–causes and consequences. Psychiatr Danub. 2010;22:540–3. 21169895

[34] Franz L Carter T Leiner AS. Stigma and treatment delay in first-episode psychosis: a grounded theory study. Early Interv Psychiatry. 2010;4:47–56. 20199480 10.1111/j.1751-7893.2009.00155.x PMC2860376

[35] Norman RM Mallal AK Manchanda R. Does treatment delay predict occupational functioning in first-episode psychosis? Schizophr Res. 2007;91:259–62. 17291725 10.1016/j.schres.2006.12.024

[36] Vera I Rezende L Molina V. Clozapine as treatment of first choice in first psychotic episodes. What do we know? Actas Esp Psiquiatr. 2012;40:281–9. 23076611

